# Supplementary material for: A Fe3N/carbon composite electrocatalyst for effective polysulfides regulation in room-temperature Na-S batteries
Source: Nat Commun. 2021 Nov 3;12:6347. doi: 10.1038/s41467-021-26631-y (PMC8566531; doi:10.1038/s41467-021-26631-y)
Supplement: Supplementary file 1 — Supplementary information [file 41467_2021_26631_MOESM1_ESM.pdf]

Supplementary Information

A Fe_3N/carbon composite electrocatalyst for effective polysulfides regulation in room-temperature Na-S batteries

Yuruo Qi[1], Qing-Jie Li[3], Yuanke Wu[1], Shu-juan Bao[1], Changming Li[1], Yuming Chen[2,3]*, Guoxiu Wang[4]*, Maowen Xu[1]*

[1] *Key Laboratory of Luminescence Analysis and Molecular Sensing, Ministry of Education, Faculty of Materials and Energy, Southwest University, Chongqing 400715, PR China*

[2] *College of Environmental Science and Engineering, Fujian Normal University, Fuzhou 350007, PR China*

[3] *Department of Nuclear Science and Engineering, Massachusetts Institute of Technology, Cambridge, Massachusetts 02139, United State*

[4] *Center for Clean Energy Technology, University of Technology Sydney, Sydney, NSW 2007, Australia*

These authors contribute equally: Yuruo Qi, Qing-Jie Li

Correspondence: yumingc126@126.com; Guoxiu.Wang@uts.edu.au; xumaowen@swu.edu.cn

Supplementary Figures

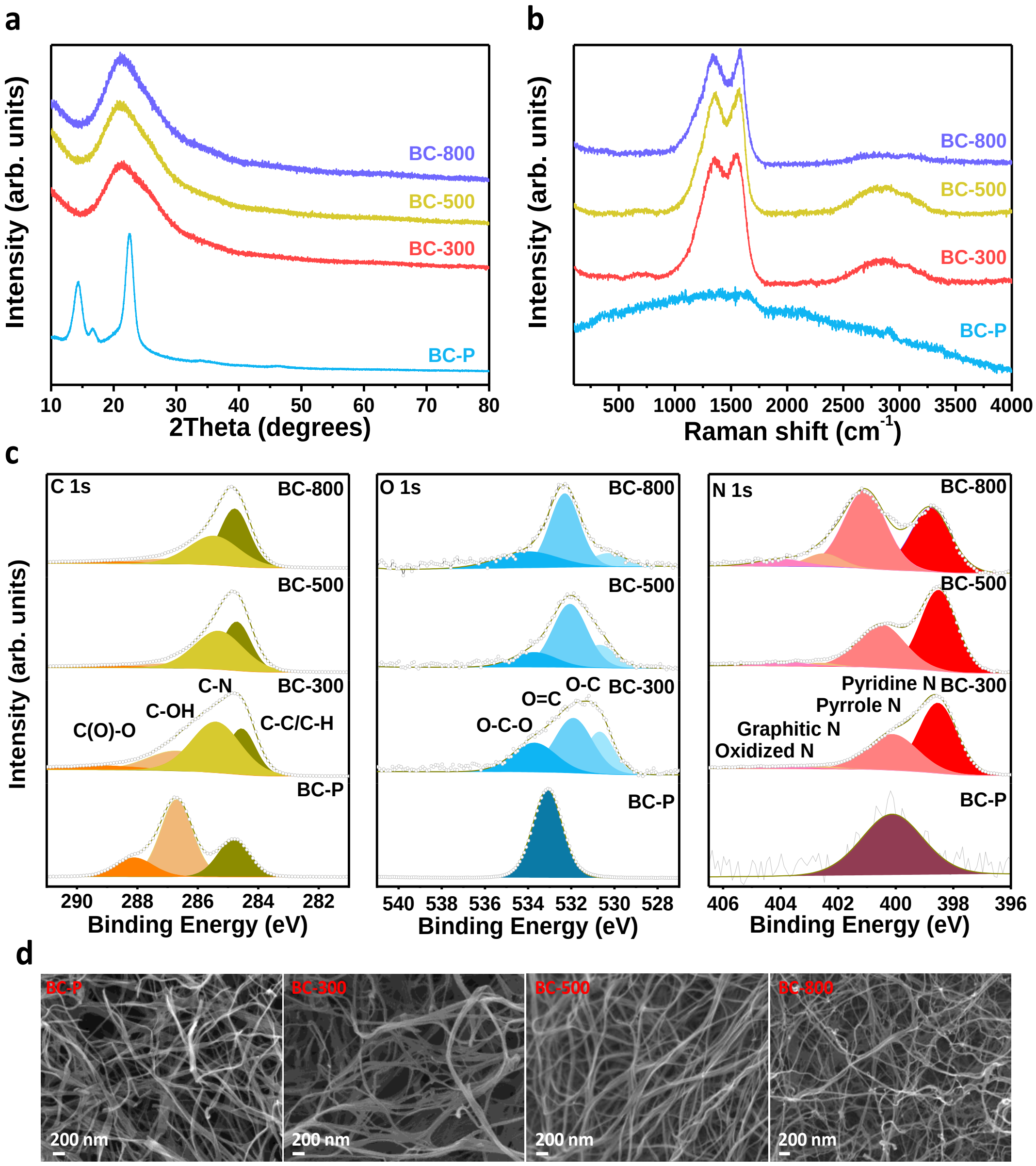

Supplementary Figure 1. XRD (**a**), Raman (**b**), XPS (**c**) and FESEM images (**d**) of the pristine BC, BC-300, BC-500 and BC-800.

To get insights on the carbonization mechanism of the bacterial cellulose, the microstructure and morphology of the product carbonized at different considerations (the pristine BC, 300 ^{o}C 1h, 300 ^{o}C 1h and 500 ^{o}C 2h, 300 ^{o}C 1h and 800 ^{o}C 2h;

abbreviated as BC-P, BC-300, BC-500 and BC-800) were characterized by XRD, Raman, XPS, FESEM and Elemental analysis, as summarized in Supplementary Figure 1 and Supplementary Table 1-2. The bare BC precursor shows three diffraction peaks at 14.3^o, 16.7^o and 22.5^o. A broad peak of amorphous carbon appears at 21.5^o after pyrolyzed at 300 ^{o}C and maintains almost unchanged with the temperature increasing. Similarly, the Raman spectra of BC-300, BC-500 and BC-800 are almost the same. FESEM images in Supplementary Figure 1d show that these composite fibers become thinner gradually with the increasing temperature.

Unlike the minor discrepancy in XRD and Raman, the surface functional groups on BC-P, BC-300, BC-500 and BC-800 are totally different. The C *1s* spectrum of the BC precursor can be fitted into three peaks of C-C/C-H, C-OH and C(O)-O at binding energies of 284.8, 286.7 and 288.1 eV. Except for these three peaks, both BC-300, BC-500 and BC-800 possess strong bonds ascribed to C-N groups at around 285.4 eV. It is obvious that the proportion of C-N bonds compared with C-C/C-H declines as the increase of temperatures. In the O *1s* spectrum, three bands of O-C, O=C and O-C-O are detected at 530.7, 531.9 and 533.7 eV for BC-300. As the rise of temperature, the amount of O=C species increases while those of O-C and O-C-O decrease dramatically. Finally, the O=C groups occupy about 63.4wt.% in BC-800, becoming the main part of oxygen. It has been reported that O=C groups can boost the affinity between the carbon anode and sodium ions, a favorable feature for performance enhancement. A weak N *1s* signal is observed for the pristine BC even before NH_3 treatment. The small amount of “intrinsic” nitrogen atoms come from the residual

nitrogen-containing compounds left by the culture media and secretions. At 300 ^{o}C, pyridinic and pyrrolic N are two main N functional groups. As temperature rises, part of the pyridinic N converts into pyrrolic, graphitic and oxidized N. Finally, the total content of pyrrdinic and pyrollic N occupies 88.1% in BC-800, which can strongly bond to polysulfides and increase electronic conductivity. In a whole, as concluded in Supplementary Table 2, the content of N/O is the highest at 300 ^{o}C and it decreases with the rise of temperature.

To accurately examine the variation of elemental content, elemental analysis was employed. As listed in Supplementary Table 1, the pristine BC consists of 46.86wt.% C, 0.12wt.% N, 46.37wt.% O and 6.65wt.% H. Apparently, a small amount (0.12wt.%) of nitrogen is existed in BC-P, in line with the XPS result. When treated at 300 ^{o}C, most of the O/H volatilizes and a large amount of N (31.45wt.%) is doped. As increase of temperature, the amount of N/O/H reduces and the content of C increases remarkably. Finally, the C, N, O and H in BC-800 are 89.77wt.%, 6.45wt.%, 2.56wt.% and 1.22wt.%, respectively. The elemental content and variation trend versus temperatures obtained by elemental analysis are highly consistent with the above-mentioned XPS results. In a whole, a carbon matrix containing a certain amount of O=C, pyridinic N, pyrrolic N with little H is obtained at 800 ^{o}C.

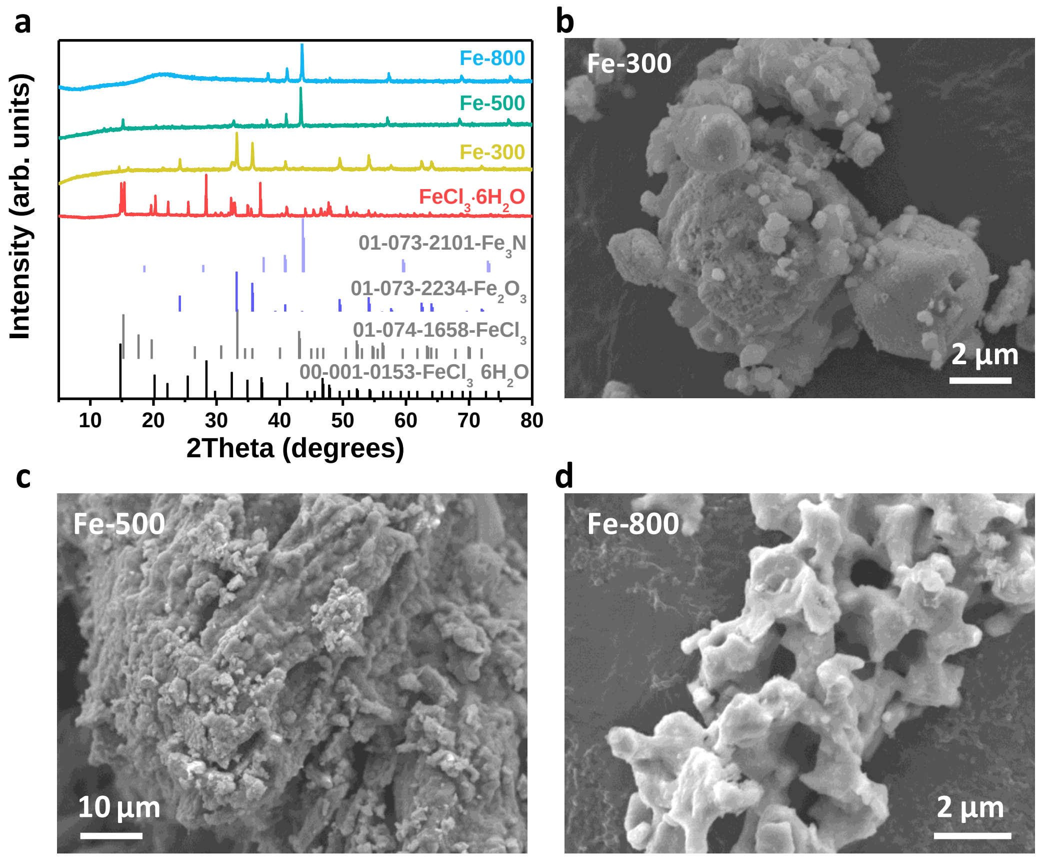

Supplementary Figure 2. XRD patterns (**a**) and FESEM images (**b-d**) of the $FeCl_3$ $6H_2O$ starting material, Fe-300, Fe-500 and Fe-800.

To understand the growth mechanism of Fe_3N, $FeCl_3$ $6H_2O$ was sintered in NH_3 atmosphere at different conditions (300 ^oC 1h, 300 ^oC 1h and 500 ^oC 2h, 300 ^oC 1h and 800 ^oC 2h; abbreviated as Fe-300, Fe-500, Fe-800). XRD patterns for products obtained at different conditions were shown in Supplementary Figure 2a. Note that there is a few of $FeCl_3$ in the starting material due to the loss of crystal water. After heated at 300 ^oC, most of the sample converts into Fe_2O_3 with a small proportion of unreacted $FeCl_3$ $6H_2O$. The product obtained at 300 ^oC presents as large particles. In the case of 500 ^oC, the Fe_3N dominates while a small amount of Fe_2O_3, $FeCl_3$, $FeCl_3$ $6H_2O$ could still be discovered. The product obtained at 500 ^oC is composed of

several small particles. Finally, a porous structured Fe_3N is obtained at 800 ^oC. In a whole, the $FeCl_3$ $6H_2O$ turns into Fe_2O_3 at the low temperature range (around 300 ^oC) and then fully converts into Fe_3N at 800 ^oC.

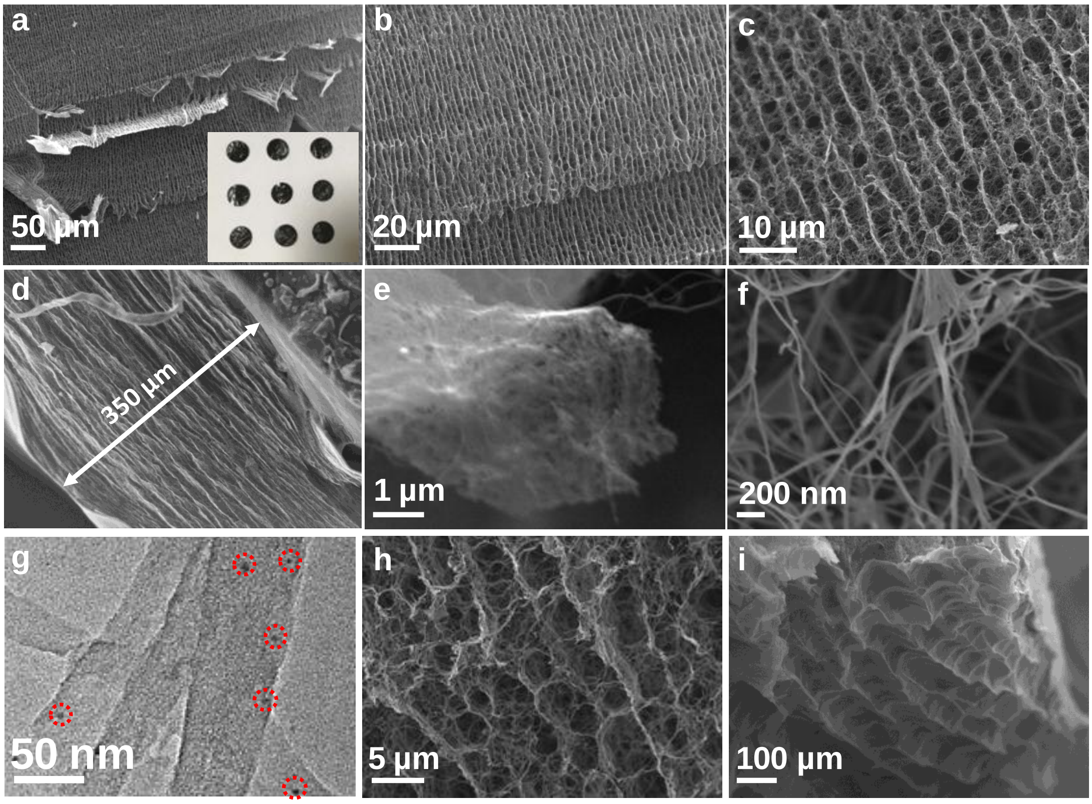

Supplementary Figure 3. FESEM (a-f, h-i) and TEM (g) images of Fe_3N-NMCN.

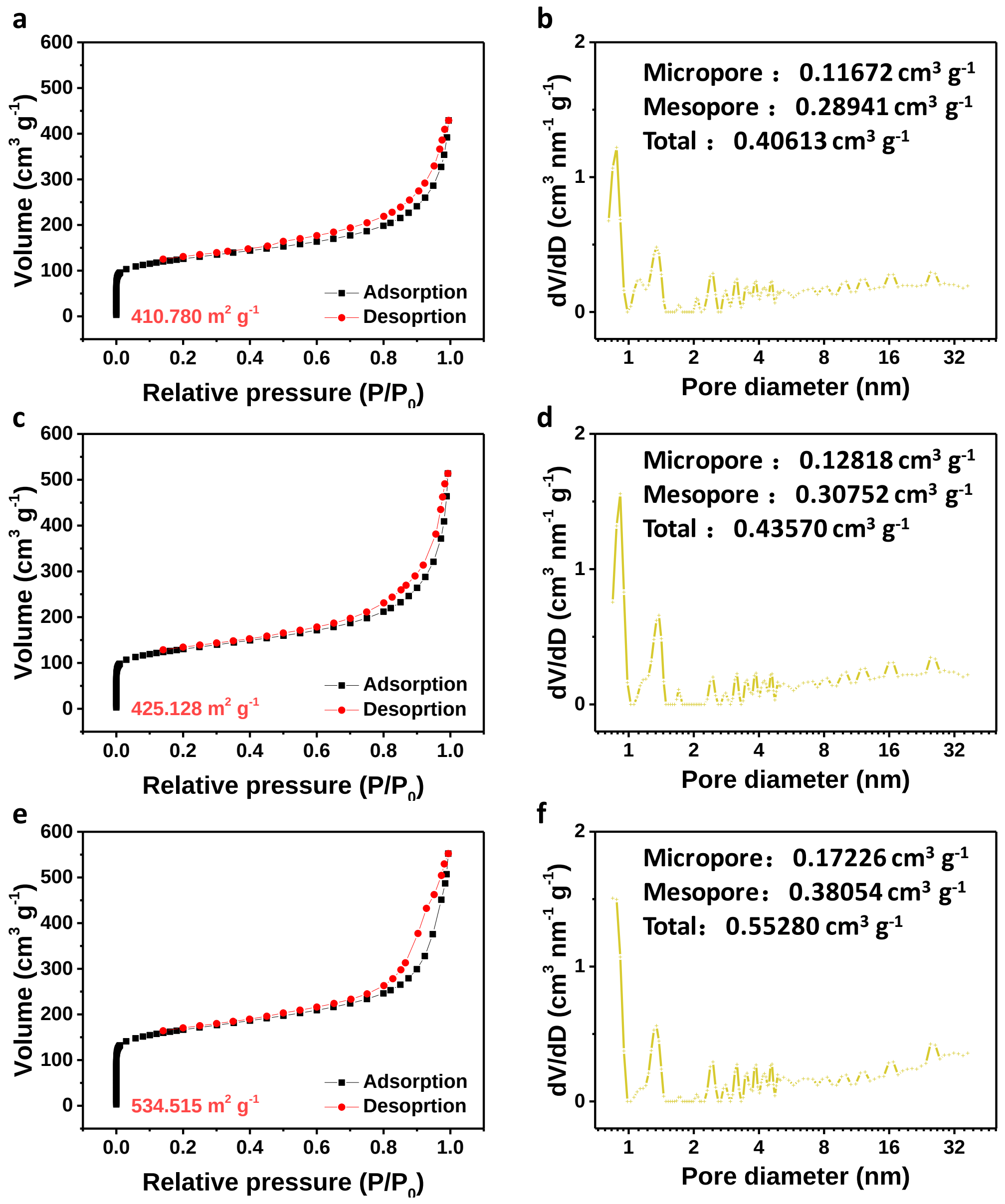

Supplementary Figure 4. Adsorption/desorption isotherms and pore-size distribution curves of BC-Ar (**a-b**), BC-NH_3 (**c-d**) and Fe_3N-NMCN (**e-f**).

The adsorption/desorption isotherms and pore-size distribution curves of BC-Ar, BC-NH_3 and Fe_3N-NMCN were compared in Supplementary Figure 4. The BET surface area, total pore volume, micropores volume and mesopores volume were summarized in Supplementary Table 3. As displayed, the Fe_3N-NMCN sample shows

the highest BET surface area of 535 m^2 g^{-1} while those of BC-Ar and BC-NH_3 are 411 and 425m^2 g^{-1}.

The total pore volume, micropores volume and mesopores volume for Fe_3N-NMCN are 0.55, 0.17, 0.38 cm^3 g^{-1}, respectively. The ratio of micropores is 31.0%. As for BC-NH_3 and BC-Ar, both the total pore volume and the proportion of micropores are reduced. It is likely that the heat treatment in NH_3 and loading of Fe_3N can introduce some mesopores and then enhance surface areas, which is beneficial for the immobilization of sulfur thus enabling a high-loading cathode.

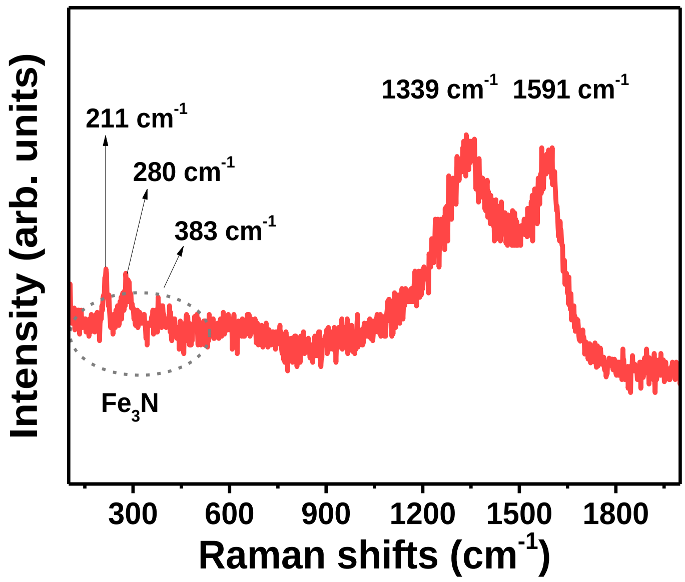

Supplementary Figure 5. Raman spectrum of Fe_3N-NMCN.

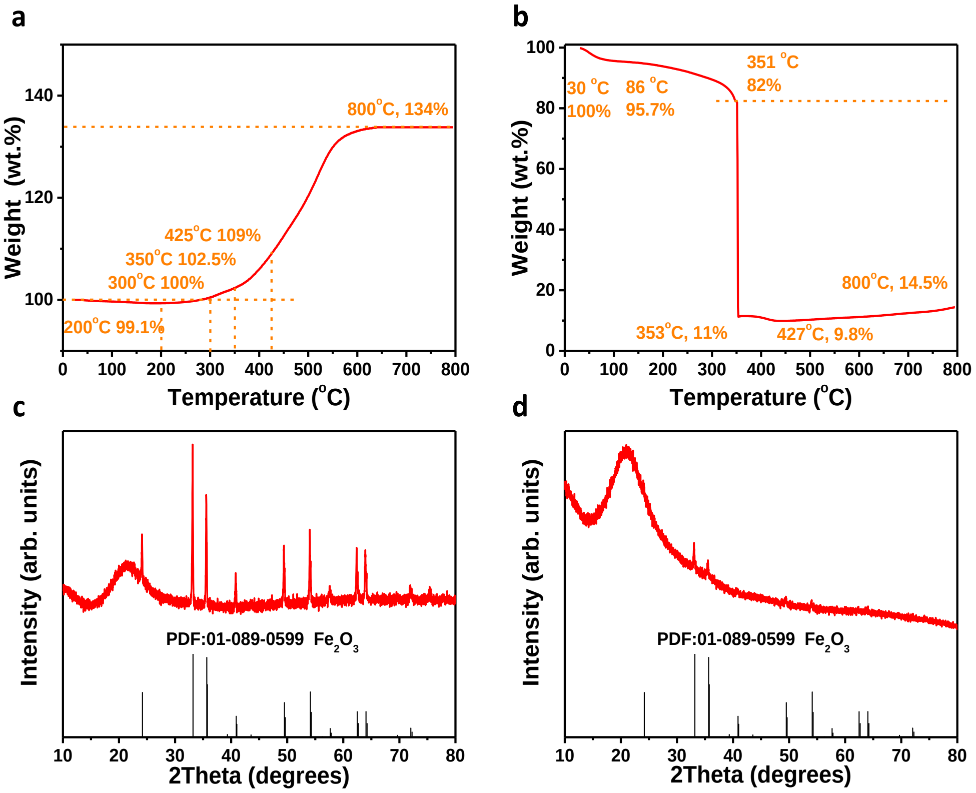

Supplementary Figure 6. Calculation of Fe_3N content in the Fe_3N-NMCN composite. **a-b** TGA tests of the bare Fe_3N powder (**a**) and the Fe_3N-NMCN composite (**b**). **c-d** XRD patterns of the final products after TGA tests from the bare Fe_3N powder (**c**) and the Fe_3N-NMCN composite (**d**).

TGA tests were employed to detect the loading of Fe_3N in the Fe_3N-NMCN composite. To clarify the transformation process of Fe_3N, TGA tests of the bare Fe_3N powder and Fe_3N-NMCN in O_2 atmosphere were conducted (Supplementary Figure 6a-b). Moreover, the phase information of the final products after TGA tests was detected by XRD (Supplementary Figure 6c-d). The bare Fe_3N powder was obtained through the heat treatment of $FeCl_3 \cdot 6H_2O$ at 300 ^{o}C for 1 h and then 800 ^{o}C for 2 h.

As displayed in Supplementary Figure 6c-d, both products obtained after TGA tests are Fe_2O_3 (159.6 g mol^{-1}). When we assume that there is 1 mol Fe_3N (181.4 g

mol^{-1}), there should be 1.5 mol Fe_2O_3 after the TGA test since the mole number of Fe is constant. During this process, the weight increment is 132.0wt.% (=159.6×1.5/181.4). This result is highly consistent with the TGA curve of the bare Fe_3N powder in Supplementary Figure 6a, where the weight increase between room temperature and 800 ^{o}C is about 134wt.% and the main weight variation is in the range of 350 and 800 ^{o}C.

Therefore, it can be deduced that the weight loss (89wt.%) of Fe_3N-NMCN before 353 ^{o}C (Supplementary Figure 6b) is exclusively originated from the consumption of carbon matrix in Fe_3N-NMCN and is not related to Fe_3N, while the weight increment (132wt.%=14.5wt.%/11wt.%) between 353 and 800 ^{o}C is only from the oxidation of Fe_3N to Fe_2O_3. From above analyses, it has been determined that the weight percentage of Fe_3N in Fe_3N-NMCN is 11wt%.

Elemental analysis (Supplementary Table 4) was conducted to obtain the total content of C, N, H and O in these composites. As demonstrated, the doped N in BC-NH_3 and BC-Ar are 6.45wt.% and 0.34wt.%, respectively. Note that the small amount of “intrinsic” nitrogen atoms in BC-Ar comes from the residual nitrogen-containing compounds left by the culture media and secretions. In addition, Supplementary Table 4 shows that the weight ratio of C, N, H, O in the Fe_3N-NMCN are 61.50wt.%, 10.21wt.%, 1.48wt.% and 12.38wt.%, respectively. According to the above TGA result, the loading of Fe_3N is 11wt.% and thus the N from the Fe_3N is calculated to 0.85wt.% (=11×14/(56×3+14)=0.85wt.%). Thus, the doped N in the

composite is 9.36wt.% (10.21-0.85=9.36wt.%). In a whole, the N doped in BC-Ar, BC-NH_3 and Fe_3N-NMCN are 0.34wt.%, 6.45wt.% and 9.36wt.%, respectively.

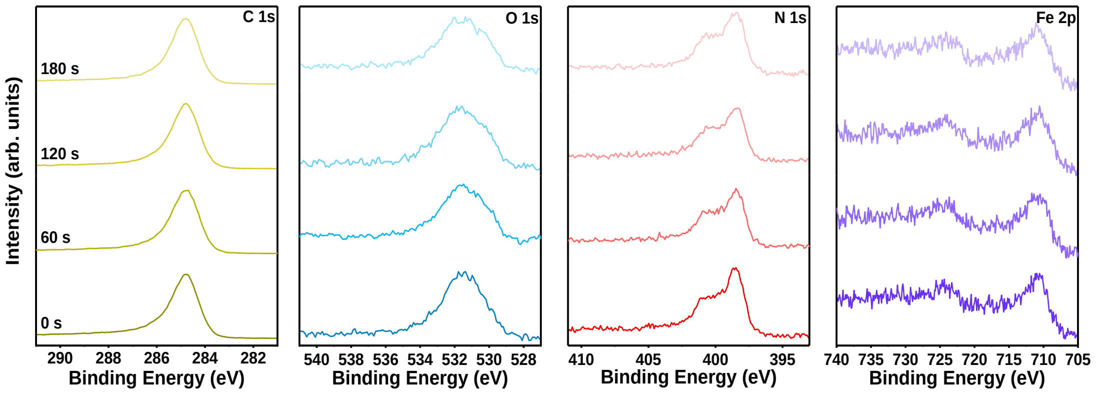

Supplementary Figure 7. XPS depth profiles after 0, 60, 120, 180 s of sputtering.

The TEM images in Figure 1d-e and Supplementary Figure 3 show that the Fe_3N quantum dots are coated with a carbon fiber. To further detect whether the N-doping and Fe_3N present on carbon surface or are embedded in the bulk of the material, XPS depth profiling results after 0, 60, 120, 180 s of sputtering were collected as Supplementary Figure 7. As displayed, all elements (C, O, N, Fe) are existed throughout the sample. Moreover, the peak shape maintains unchanged. The above results demonstrate that both the N-doping and Fe_3N are distributed uniformly through the whole sample.

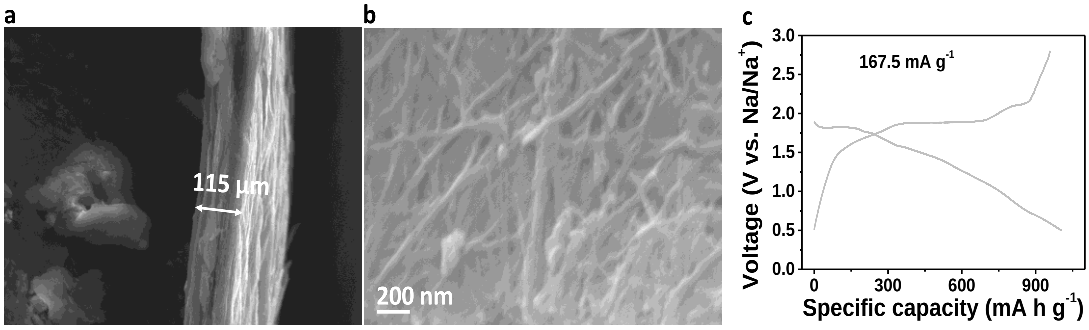

Supplementary Figure 8. FESEM images (a-b) and voltage-capacity curve (c) of the compact S@Fe_3N-NMCN electrode obtained through a calendaring-infiltration strategy.

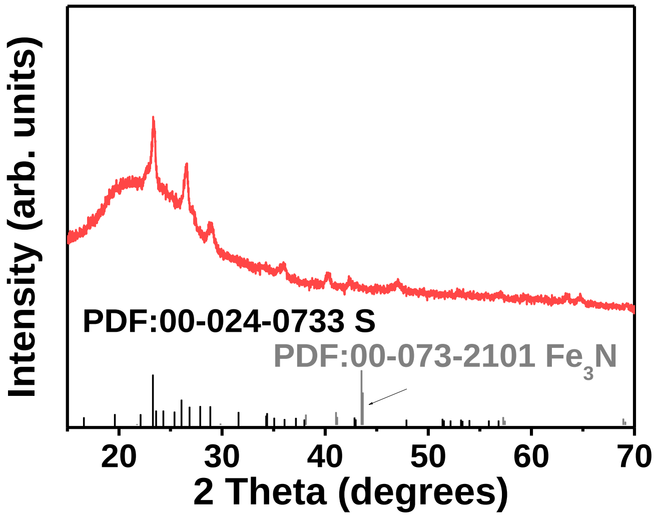

Supplementary Figure 9. XRD pattern of S@Fe_3N-NMCN.

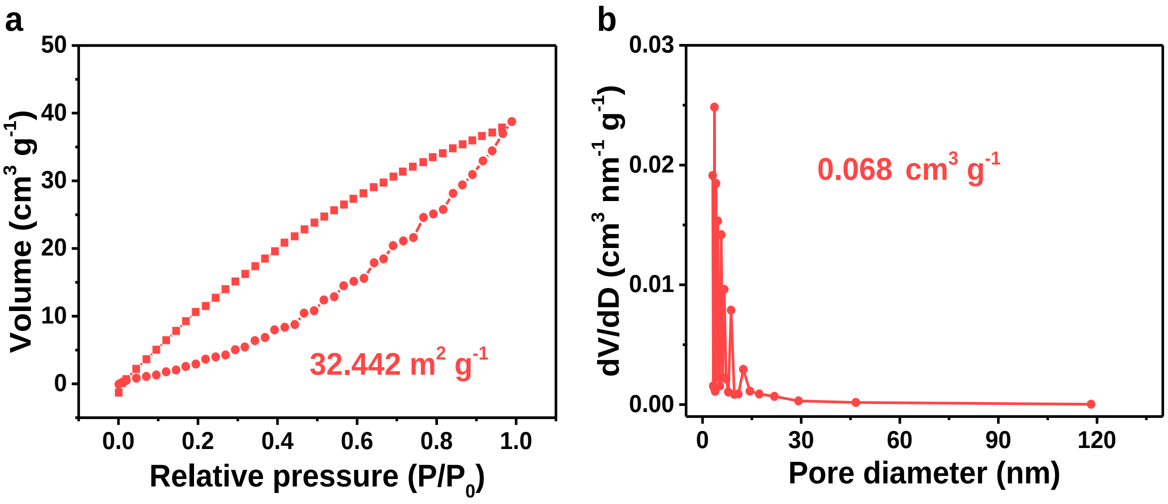

Supplementary Figure 10. N_2 adsorption/desorption curves (**a**) and corresponding pore-size distribution curve (**b**) of S@Fe_3N-NMCN.

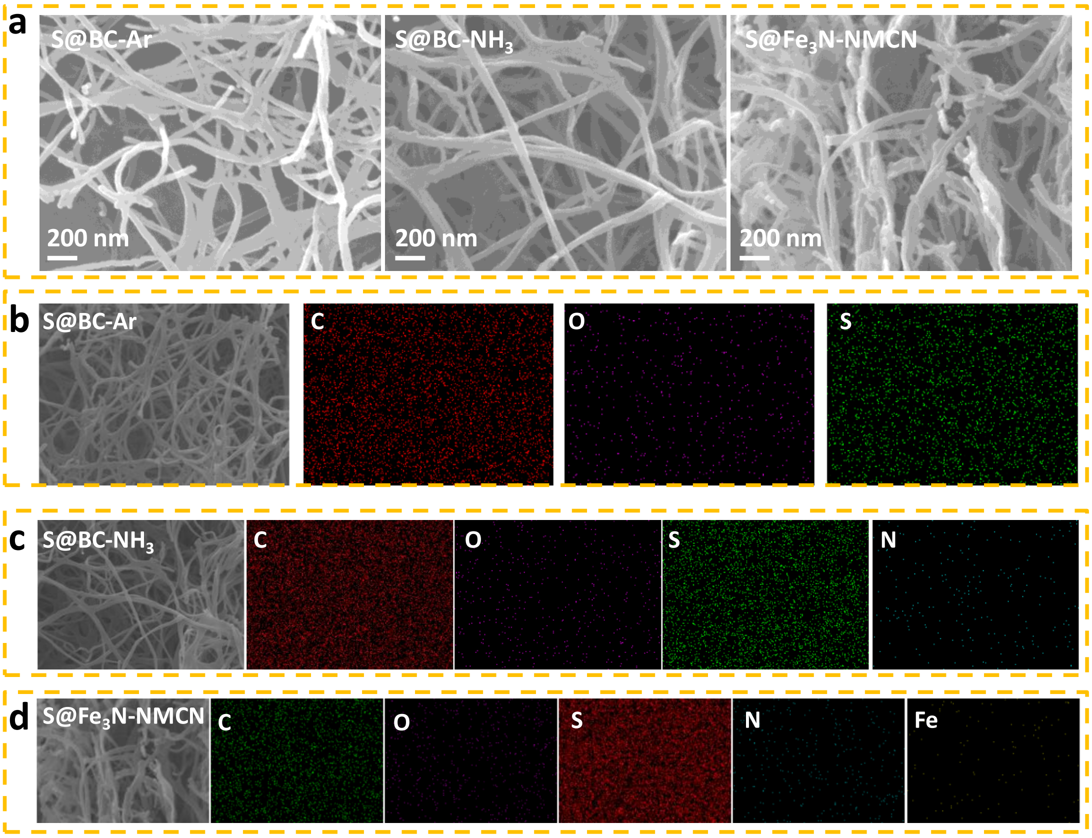

Supplementary Figure 11. FESEM and corresponding EDS mapping images of S@BC-Ar, S@BC-NH_3 and S@Fe_3N-NMCN, respectively.

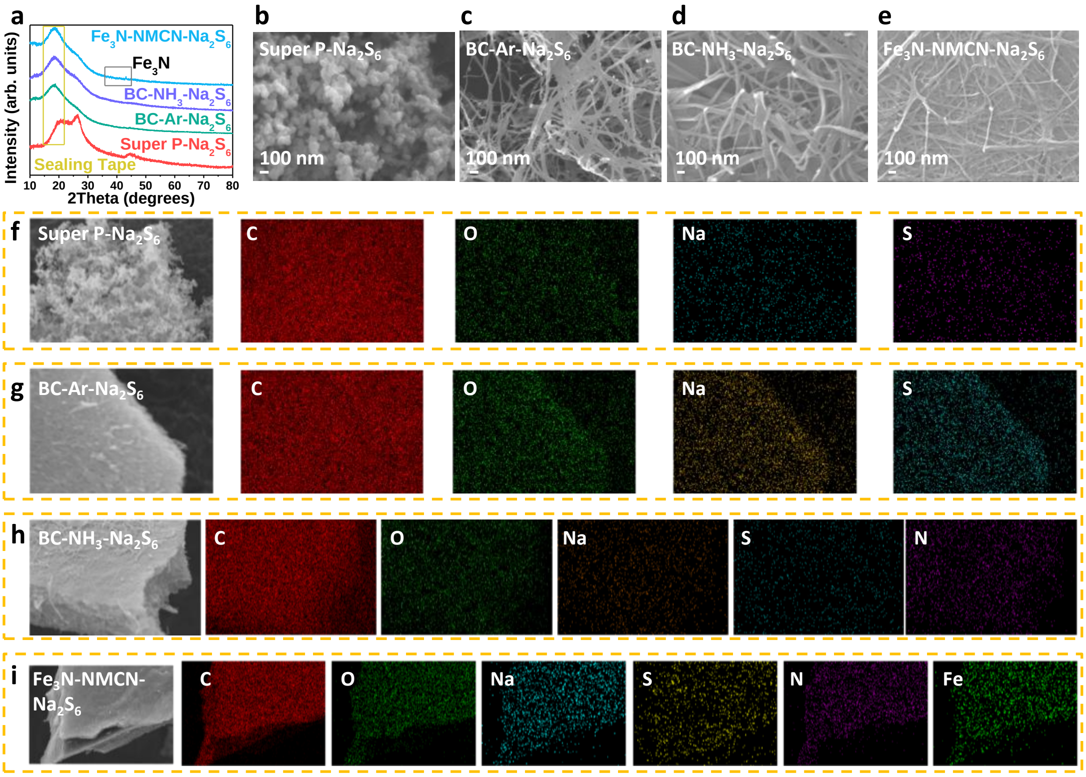

Supplementary Figure 12. XRD (**a**), FESEM images (**b-e**) and EDS mapping images (**f-i**) of Super P-Na_2S_6, BC-Ar-Na_2S_6, BC-NH_3-Na_2S_6 and Fe_3N-NMCN-Na_2S_6, respectively.

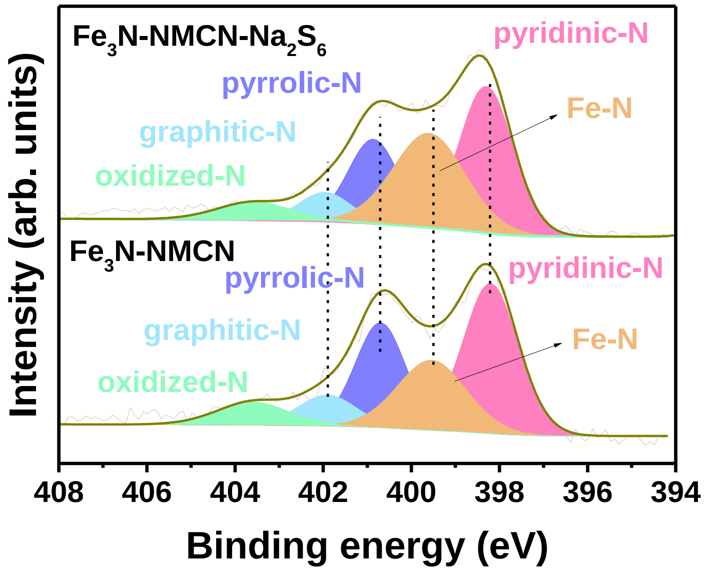

Supplementary Figure 13. N *1s* XPS spectra of Fe_3N-NMCN and Fe_3N-NMCN-Na_2S_6.

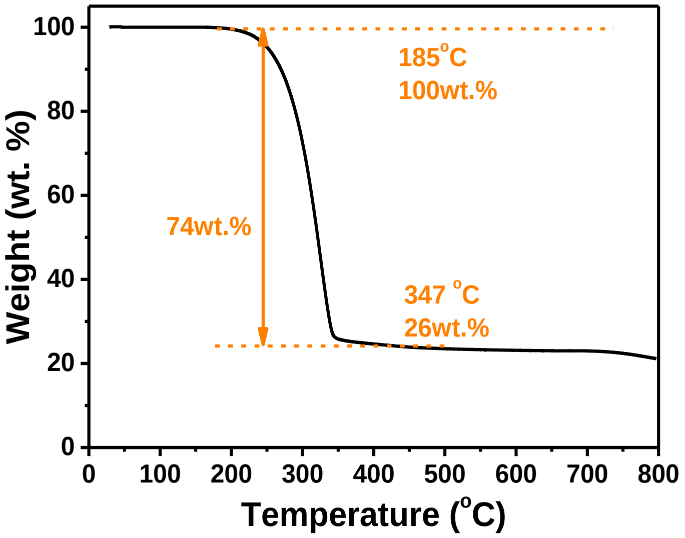

Supplementary Figure 14. TG curve of S@BC-NH_3.

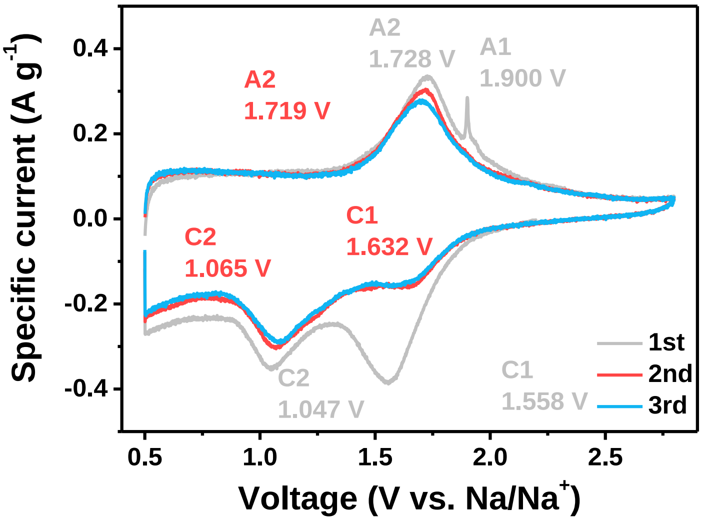

Supplementary Figure 15. First three CV curves of S@BC-NH_3 at a scan rate of 0.2 mV s^{-1}.

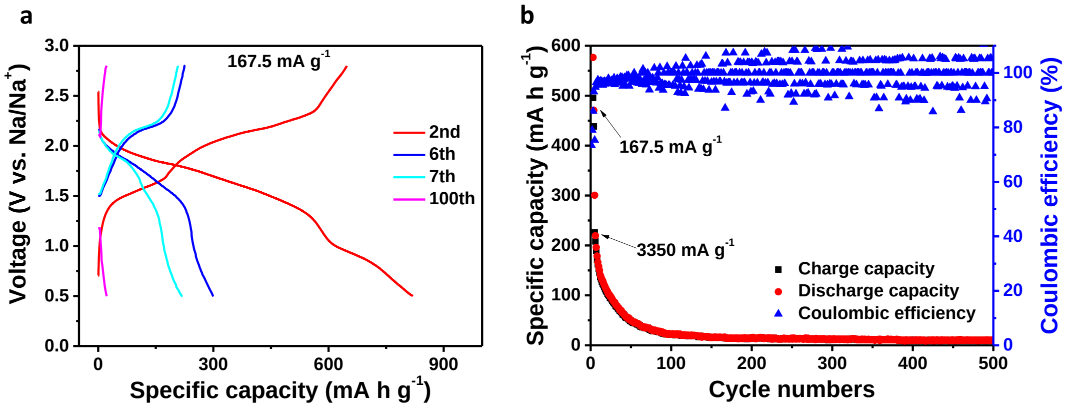

Supplementary Figure 16. Discharge/charge curves (**a**) and cycling performance (**b**) of S@Fe_3N-NMCN with 1M $NaClO_4$ in EC/DEC (1:1 by volume) electrolyte in Na-S batteries.

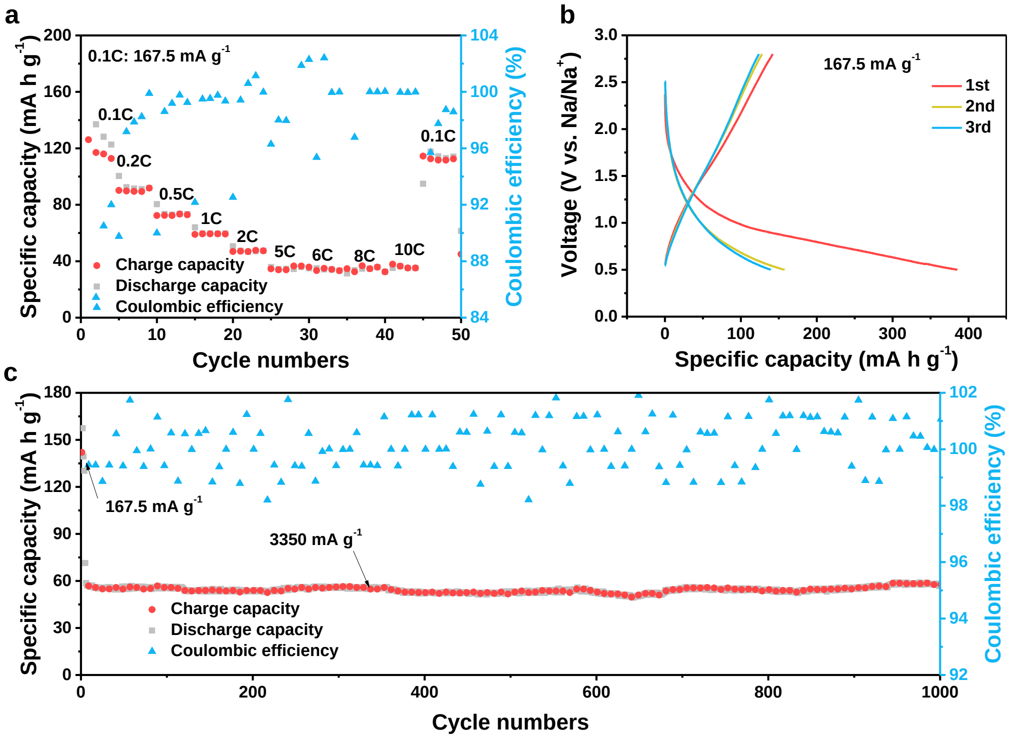

Supplementary Figure 17. Na storage performance of the Fe_3N-NMCN substrate.

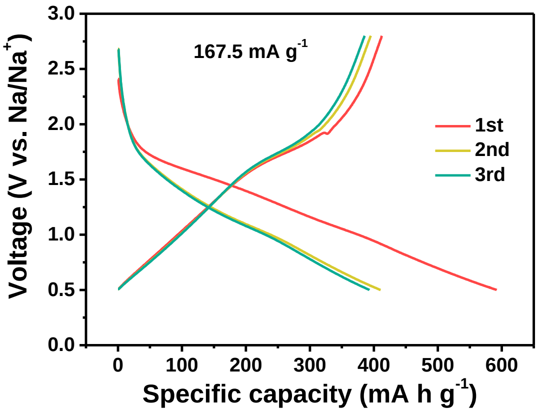

Supplementary Figure 18. Galvanostatic discharge/charge curves of S@BC-NH_3.

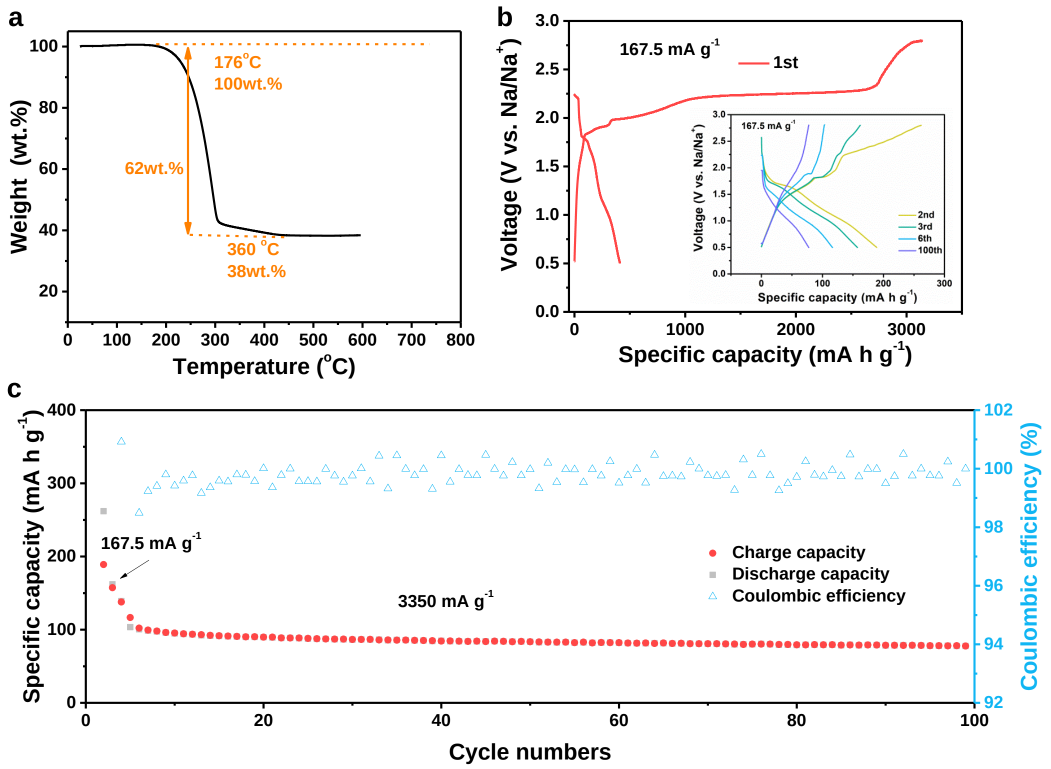

Supplementary Figure 19. Electrochemical performance of S@BC-Ar.

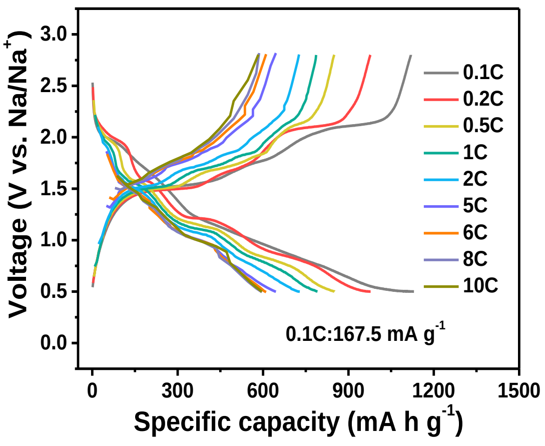

Supplementary Figure 20. Galvanostatic discharge/charge curves of S@Fe_3N-NMCN at different specific currents.

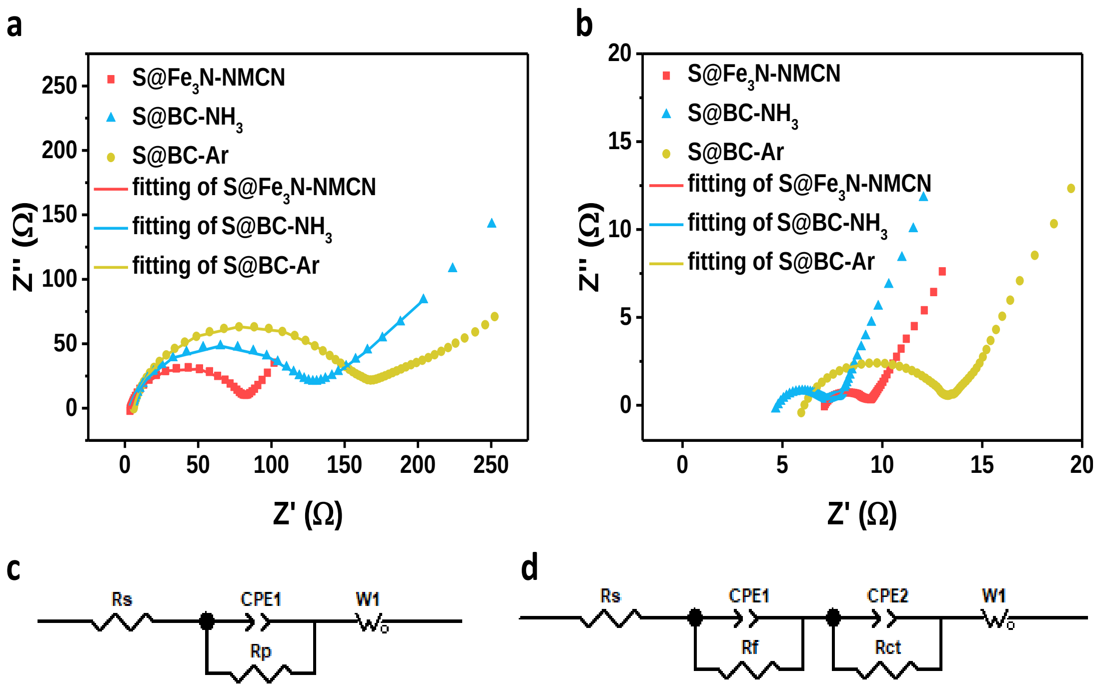

Supplementary Figure 21. EIS spectra and corresponding equivalent circuit diagrams of S@BC-Ar, S@BC-NH_3, S@Fe_3N-NMCN before (**a, c**) and after cycle (**b, d**).

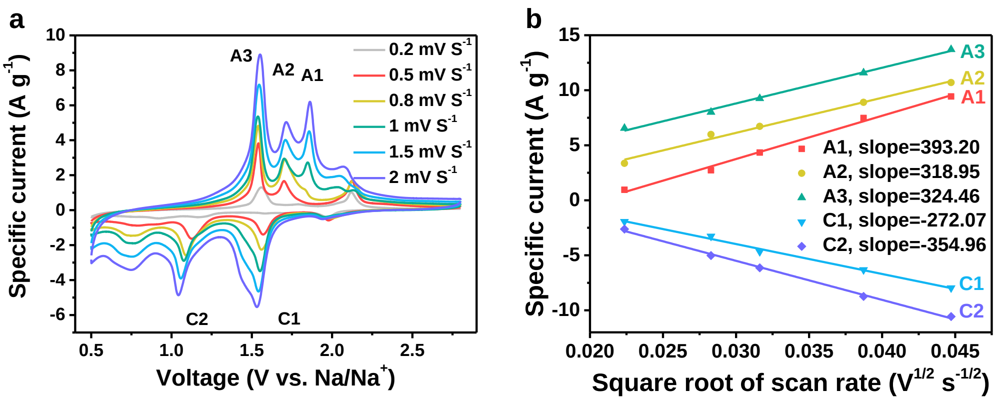

Supplementary Figure 22. (**a**) CV of S@Fe_3N-NMCN at different scan rates of 0.2, 0.5, 0.8, 1, 1.5 and 2 mV s^{-1}, (**b**) Plots of peak currents and square roots of scan rates.

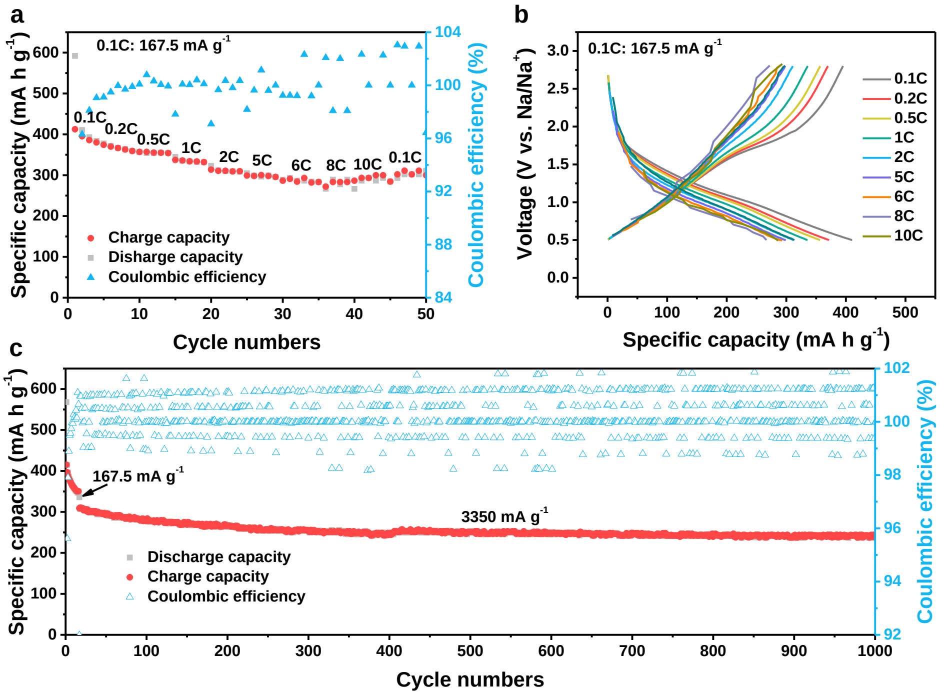

Supplementary Figure 23. Rate and cycling performance of S@BC-NH_3.

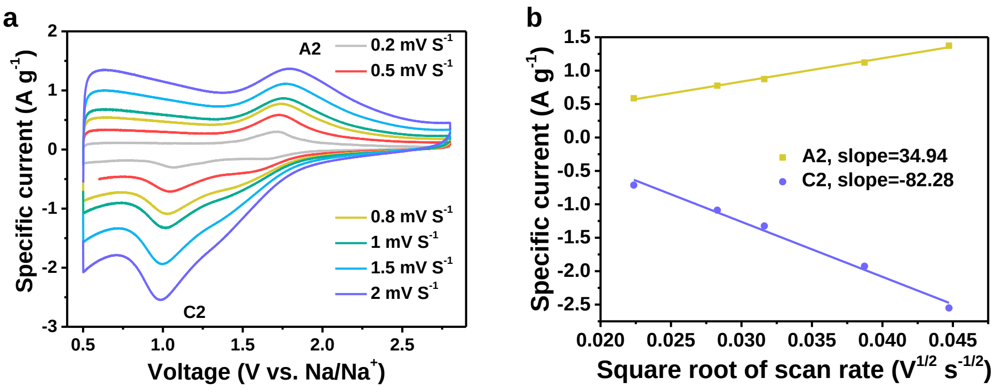

Supplementary Figure 24. (**a**) CV of S@BC-NH_3 at different scan rates of 0.2, 0.5, 0.8, 1, 1.5 and 2 mV s^{-1}, (**b**) Plots of peak currents and square roots of scan rates.

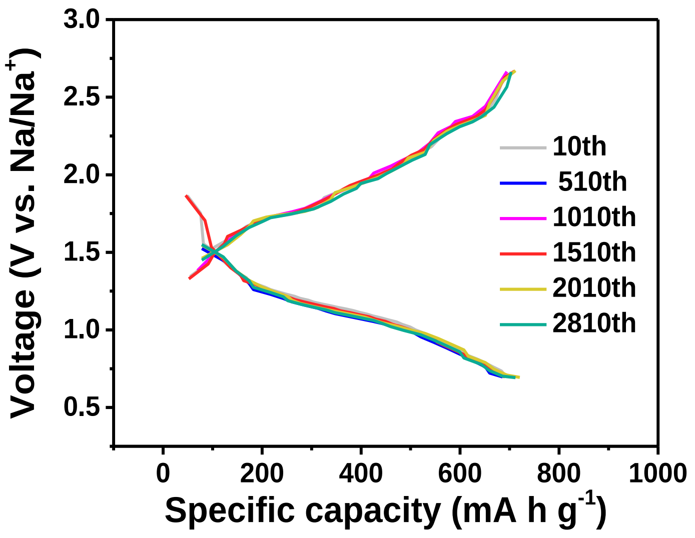

Supplementary Figure 25. Voltage-capacity curves of the S@Fe_3N-NMCN electrode during the long term cycling test at 8375 mA g^{-1} after 10 cycles at 167.5 mA g^{-1}.

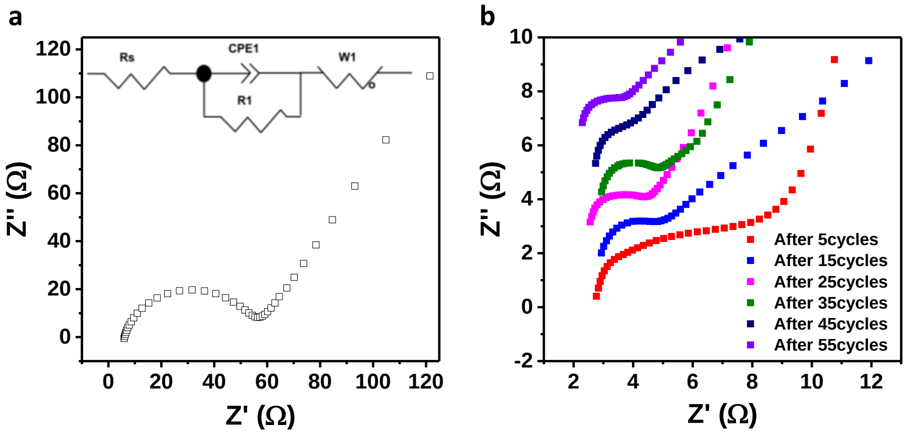

Supplementary Figure 26. EIS of batteries with S@Fe_3N-NMCN after different cycles at a specific current of 83.75 mA g^{-1}.

It is well accepted that the capacity increase in the first few cycles could stem from the activation of the electrode, as widely observed in previous reports. Furthermore, to verify the above speculation, the electrochemical impedance spectra during first 55 cycles at a specific current of 83.75 mA g^{-1} (ten times smaller than that in Figure 5f) were recorded, as shown in Supplementary Figure 26 and Supplementary Table 7. The fresh battery has a resistance of 71.25 **Ω**. The resistance declines dramatically to 6.86 **Ω** after 5 cycles (around 200 hours) and 0.89 **Ω** after 15 cycles (around 600 hours). Moreover, the value of resistance maintains unchanged in the following cycles. The evolution of EIS indicates that the proposed electrode can reach stable in about 600 hours. In addition, the ambient temperature change can also lead to the slight capacity variation. Therefore, the slight capacity variation (Figure 5f) can be due to the electrochemical activation process and the ambient temperature change.

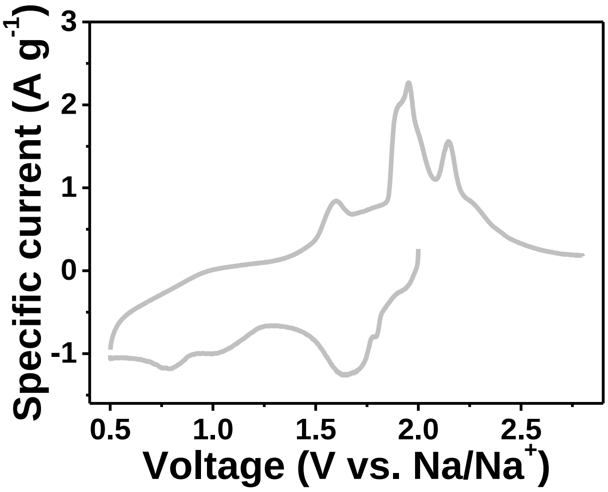

Supplementary Figure 27. CV curve for *in situ* Raman.

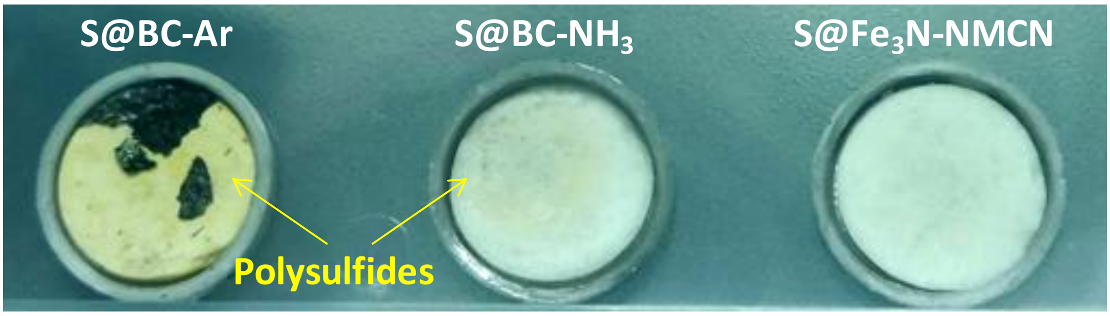

Supplementary Figure 28. Optical images of the separators from batteries with S@BC-Ar, S@BC-NH_3 and S@Fe_3N-NMCN, respectively.

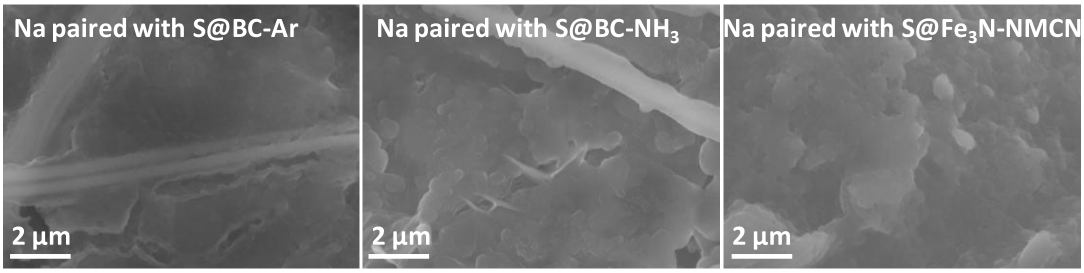

Supplementary Figure 29. FESEM images of cycled Na metal paired with S@BC-Ar, S@BC-NH_3 and S@Fe_3N-NMCN, respectively.

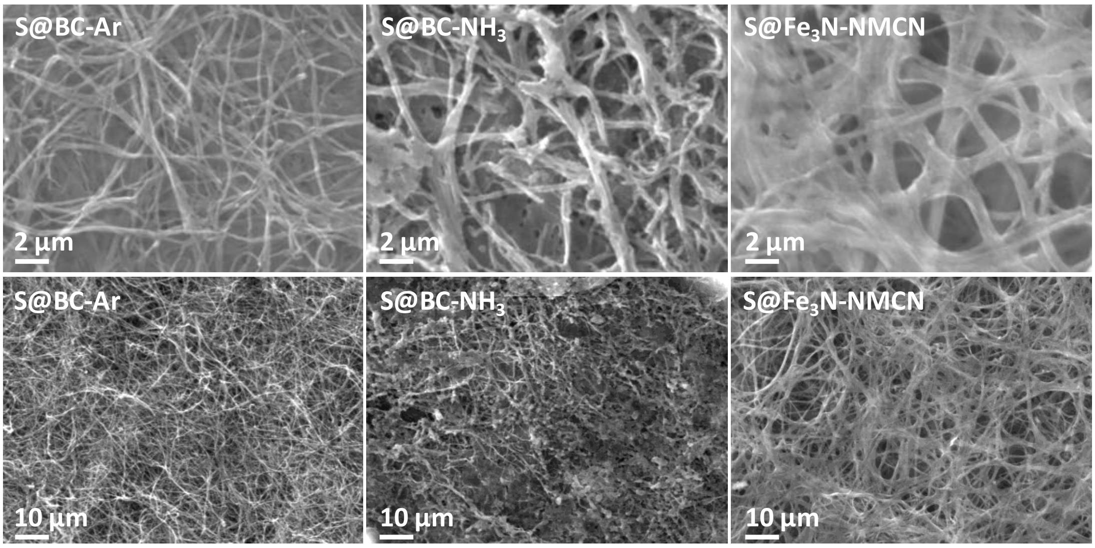

Supplementary Figure 30. FESEM images of cycled S@BC-Ar, S@BC-NH_3 and S@Fe_3N-NMCN electrodes, respectively.

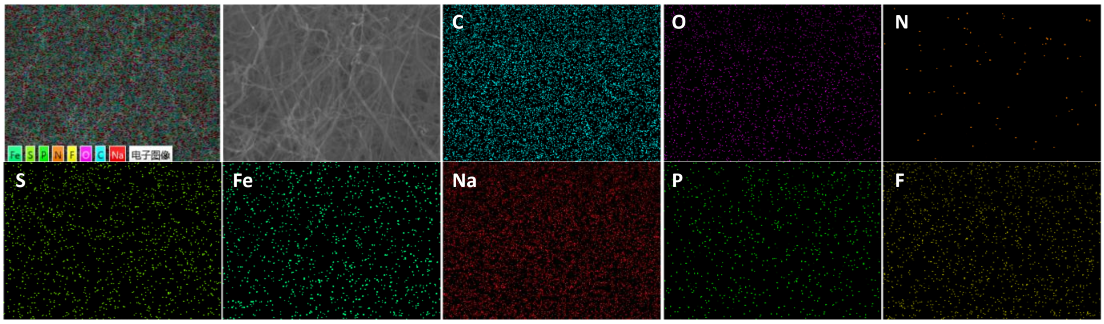

Supplementary Figure 31. EDS mapping images of cycled S@BC-Ar.

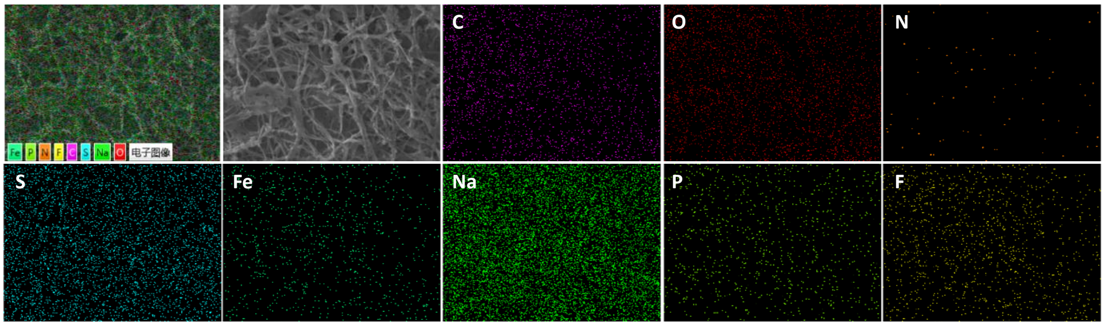

Supplementary Figure 32. EDS mapping images of cycled S@BC-NH_3.

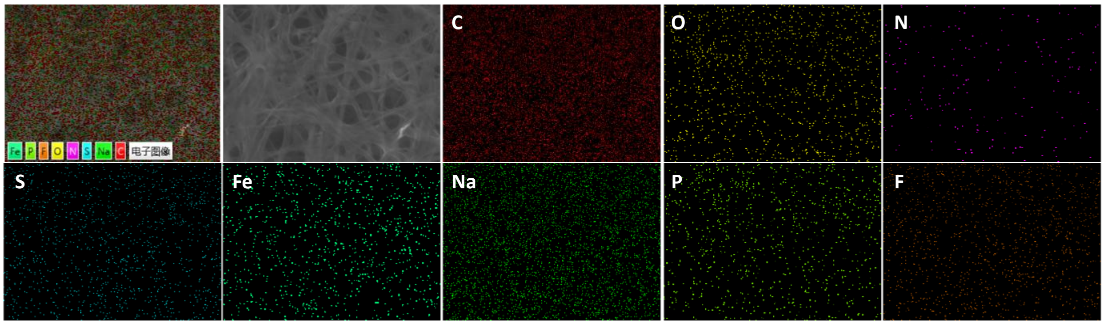

Supplementary Figure 33. EDS mapping images of cycled S@Fe_3N-NMCN.

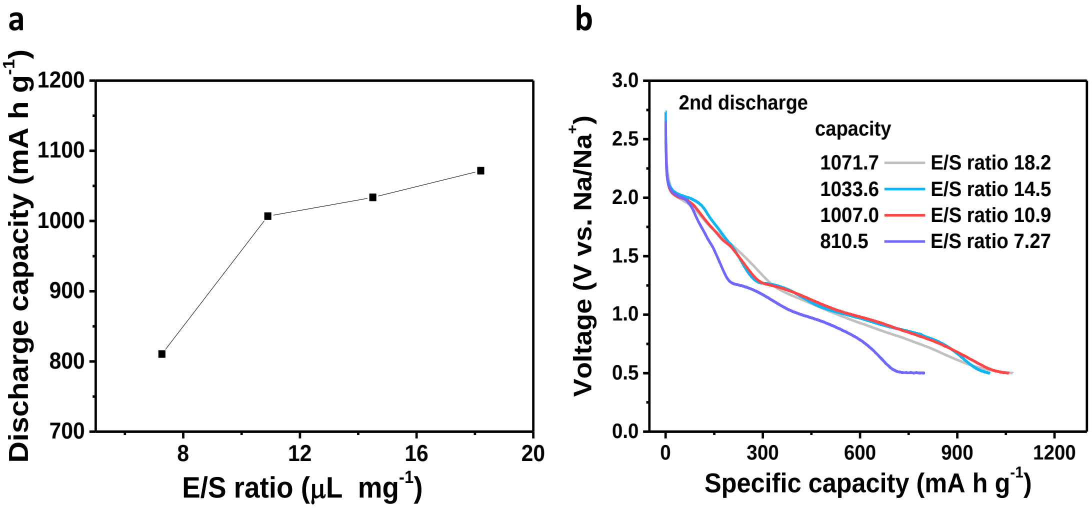

Supplementary Figure 34. Effect of electrolyte/sulfur (E/S) ratio on discharge capacity of the S@Fe_3N-NMCN electrode (**a**) and discharge profiles at different E/S ratios (**b**).

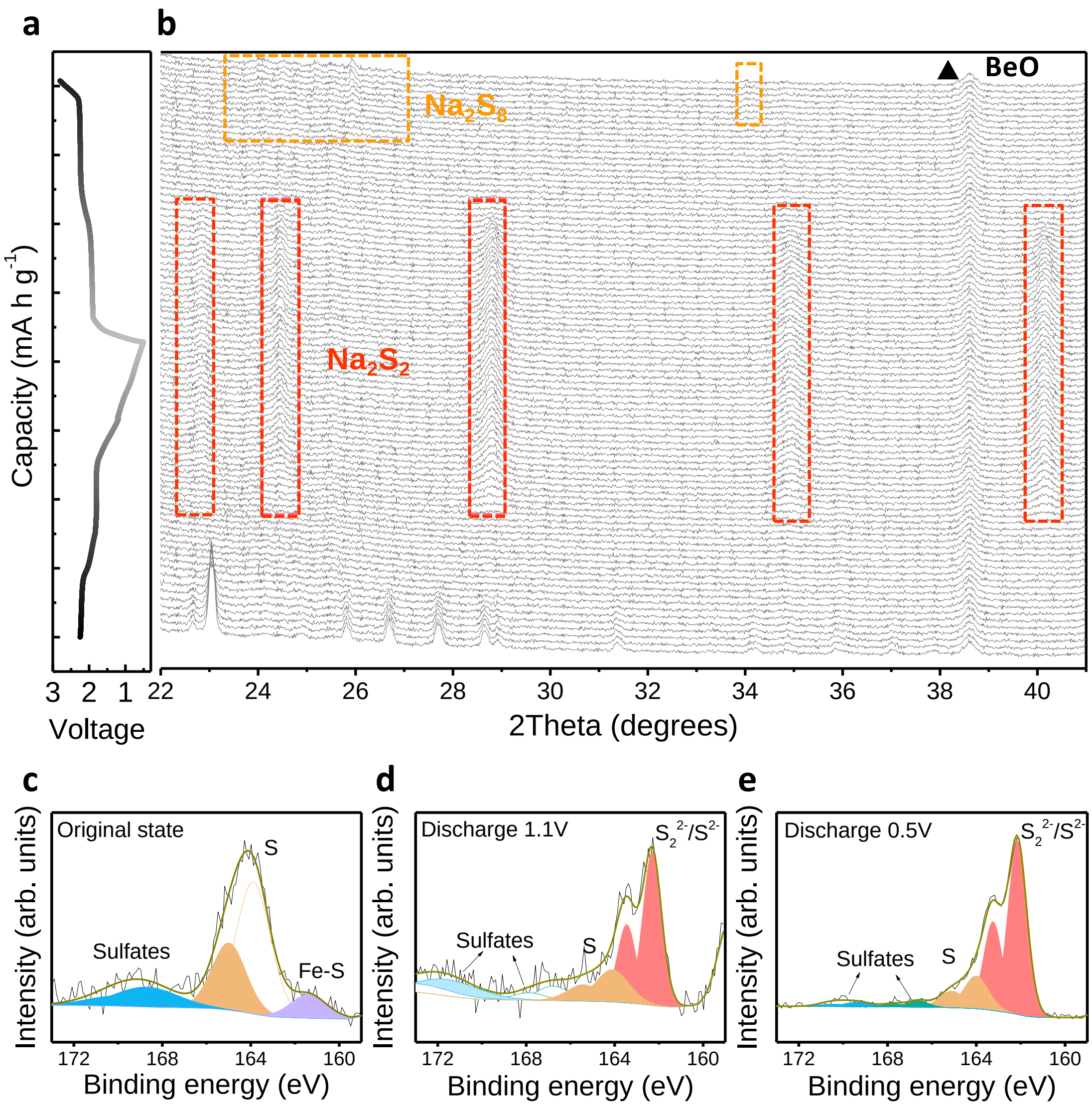

Supplementary Figure 35. *In situ* lab. XRD of the S@Fe_3N-NMCN electrode at a specific current of 167.5 mA g^{-1} with a low E/S ratio of 7.27 uL mg^{-1} in an electrochemical window of 2.8 - 0.5 V (**b**) and corresponding discharge/charge profile (**a**). **c-d** S *2p* XPS spectra at the original state (**c**), discharged to 1.1 V (**d**) and discharged to 0.5 V (**e**).

There may be three reasons for the fact that Na_2S is not presented in the *in situ* XRD pattern. First, only a few Na_2S are formed under this low E/S ratio and thus the signal of Na_2S is weak. Second, the generated Na_2S can be covered by other intermediates. Third, a conventional XRD instrument with limited resolution and power was

employed to conduct the *in situ* experiment. Therefore, diffraction peaks of Na_2S can hardly be observed by the *in situ* XRD.

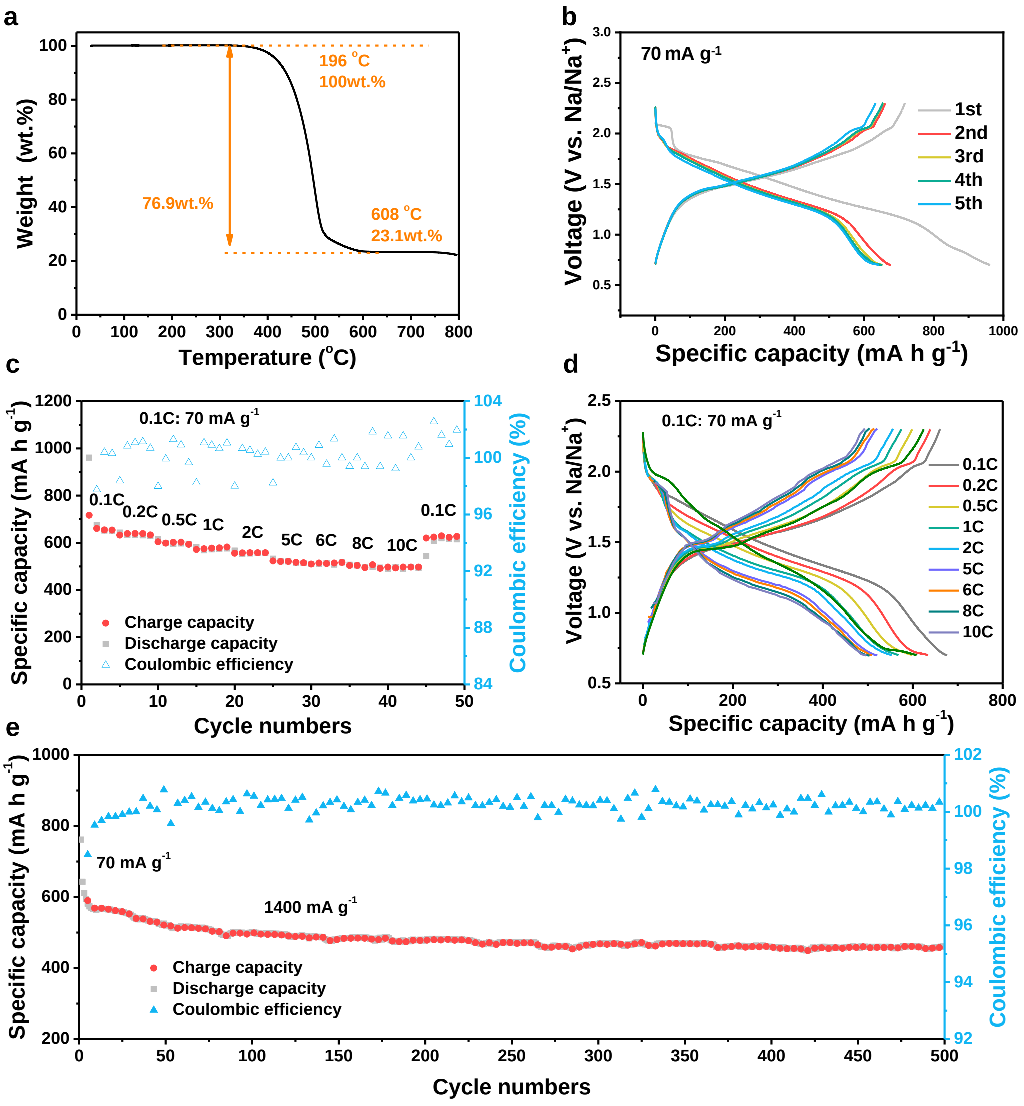

Supplementary Figure 36. Electrochemical performance of Se@Fe_3N-NMCN.

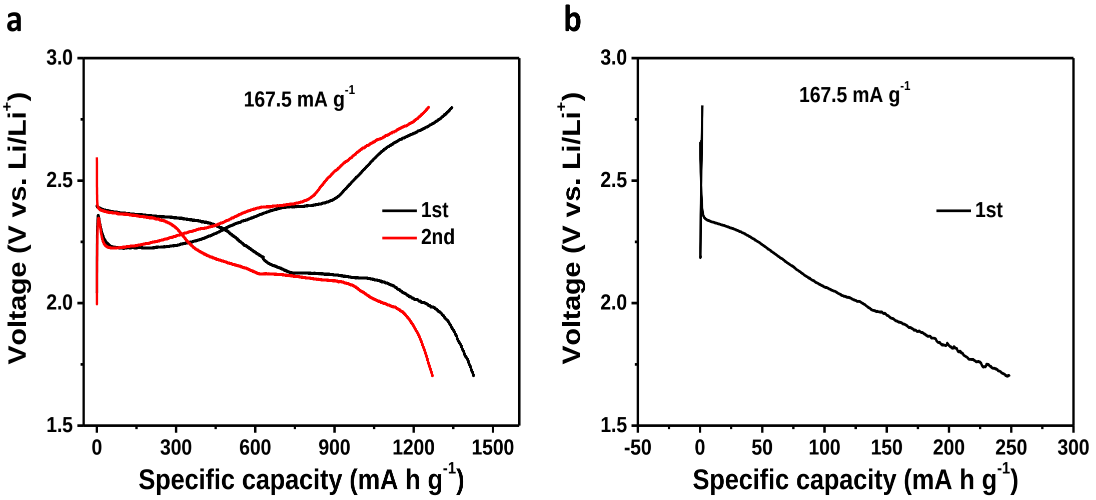

Supplementary Figure 37. Discharge/charge curves of S@Fe_3N-NMCN with 1M LiTFSI in DOL/DME (1:1 by volume) containing 0.1M $LiNO_3$ additive (**a**) and 1M $LiPF_6$ in EC/DEC/EMC (1:1:1 by volume) electrolyte (**b**) in Li-S batteries.

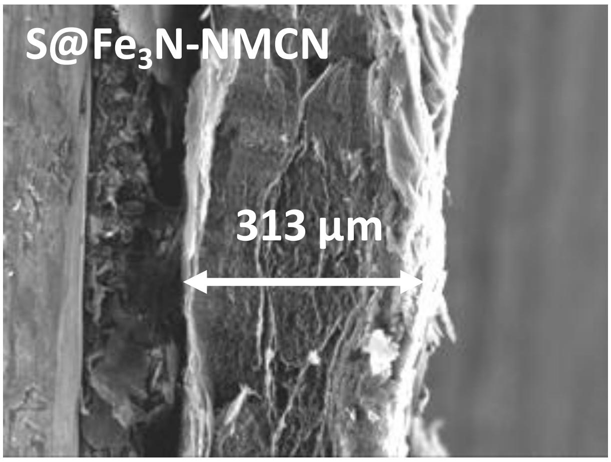

Supplementary Figure 38. Side view FESEM image of S@Fe_3N-NMCN.

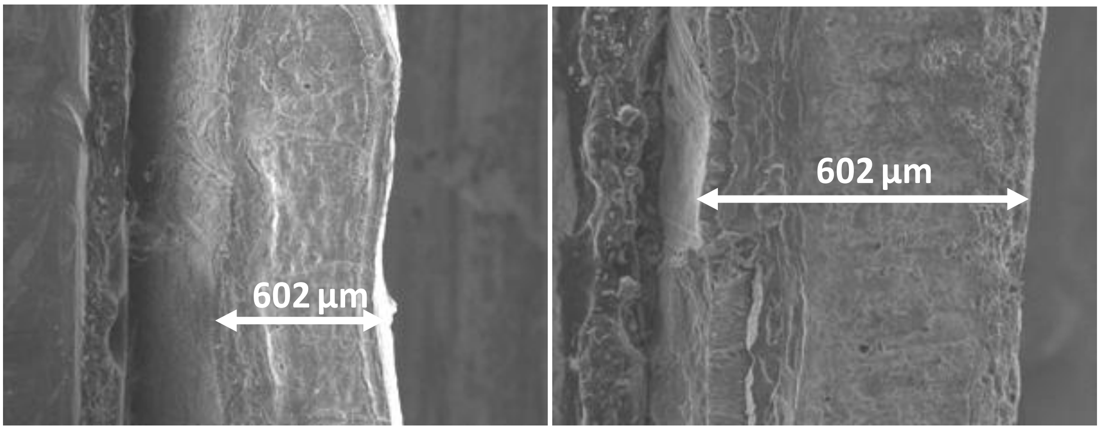

Supplementary Figure 39. Side view FESEM images of sodium foils.

Supplementary Table 1. Elemental analysis of BC-P, BC-300, BC-500 and BC-800.

weight percentage (wt.%)	BC-P	BC-300	BC-500	BC-800
C	46.86	56.83	71.85	89.77
N	0.12	31.45	20.65	6.45
O	46.37	9.07	4.76	2.56
H	6.65	2.64	2.74	1.22

Supplementary Table 2. XPS analysis of BC-P, BC-300, BC-500 and BC-800.

Weight percentage (wt.%)	BC-P	BC-300	BC-500	BC-800
C	56.07	48.26	63.37	84.12
N	0.36	40.90	26.02	7.63
O	43.57	10.84	10.61	8.25

Supplementary Table 3. BET surface areas, total pore volume, micropores volume and mesopores volume of BC-Ar, BC-NH_3 and Fe_3N-NMCN.

Sample	Surface area (m^2 g^{-1})	Total pore volume (cm^3 g^{-1})	Micropores volume (cm^3 g^{-1})	Mesopores volume (cm^3 g^{-1})	Ratio of micropores (%)
BC-Ar	411	0.41	0.12	0.29	29.3
BC-NH_3	425	0.44	0.13	0.31	29.5
Fe_3N-NMCN	535	0.55	0.17	0.38	31.0

Supplementary Table 4. Elemental analysis of BC-Ar, BC-NH_3 and Fe_3N-NMCN.

Weight percentage (wt.%)	BC-Ar	BC-NH_3	Fe_3N-NMCN
C	90.70	89.77	61.50
H	1.70	1.22	1.48
N	0.34	6.45	10.21
O	7.26	2.56	12.38

Supplementary Table 5. Fitted EIS results of batteries with S@BC-Ar, S@BC-NH_3 and S@Fe_3N-NMCN before cycle.

Sample	R_s (Ω)	R_p (Ω)
S@Fe_3N-NMCN	3.97	67.46
S@BC-NH_3	0.81	93.88
S@BC-Ar	1.39	119.70

Supplementary Table 6. Fitted EIS results of batteries with S@BC-Ar, S@BC-NH_3 and S@Fe_3N-NMCN after cycle.

Sample	R_s (Ω)	R_f (Ω)	R_{ct} (Ω)
S@Fe_3N-NMCN	7.02	2.03	1.40
S@BC-NH_3	3.60	2.10	1.53
S@BC-Ar	4.71	6.21	1.92

Supplementary Table 7. EIS fitting results of batteries with S@Fe_3N-NMCN after different cycles.

Sample	R_s (Ω)	R_1 (Ω)
Pristine	3.95	71.25
After 5 cycles	2.70	6.86
After 15 cycles	3.06	0.89
After 25 cycles	2.69	0.75
After 35 cycles	3.02	0.71
After 45 cycles	2.97	0.81
After 55 cycles	2.42	1.00

Supplementary Table 8. Comparisons of cathode materials for RT Na-S batteries

Samples	S loading	Electrolyte	Cycle performance			Rate capability
			Rate	Cycle number	Decay rate (per cycle)	
This work	85wt.%	1 M NPF_6 in DOL/DIGLYME	5C	2868	Almost no decay	1238.6, 1073.7, 936.6, 866.2, 798.6, 706.9, 671.9, 664.2, 658.4 mAh g^{-1} at 167.5, 335, 837.5, 1675, 3350, 8375, 10050, 13400, 16750 mA g^{-1}
Nickel hollow spheres concatenated by nitrogen-doped carbon fibers[1] **S@Ni-NCFs**	36%	1 M $NaClO_4$ in TEGDME	0.5C	270	0.17%	738.7, 565.6, 481.1, 401.9, 311.1, 249.8 and 181.7 mAh g^{-1} at 335, 503, 838, 1675, 3350, 5025 and 8375 mA g^{-1}
sulfurized polyacrylonitrile nanofiber[2] **SPAN**	41%	1 M $NaPF_6$ in EC/DEC	0.1C	200	0.11%	310, 210, 110, 72 mAh g^{-1} at 16.8, 168, 838, 1675 mA g^{-1}
Microporous carbon template derived from sucrose[3] **S@C**	35%	1 M $NaPF_6$ + 0.25M $NaNO_3$ in TEGDME	1C	1500	0.016%	700, 550, 470, 370 mAh g^{-1} at 168, 419, 838, 1675 mA g^{-1}
Carbon fiber cloth[4] **CFC/S**	24.4%	1.5 M $NaClO_4$+ 0.2 M $NaNO_3$ in TEGDME	0.1C	300	0.23%	491, 265, 141, 80, 48 mAh g^{-1} at 83.8, 168, 335, 838,1675 mA g^{-1}
Nitrogen doped carbon from MOF[5] **cZIF-8/S**	50%	1 M $NaClO_4$ in TEGDME	0.2C	250	0.17%	1000, 850, 650, 480, 220 mAh g^{-1} at 168, 335, 838, 1675, 3350 mA g^{-1}
Hollow carbon sphere coated with MoS_2[6] **S@HCS/MoS_2**	44%	1 M $NaClO_4$ in TEGDME	1C	1000	0.06%	1309, 856, 663, 559, 476 mAh g^{-1} at 168, 335, 838, 1675, 3350 mA g^{-1}
Free-standing carbon nanofibers embedded with cobalt nanoparticles[7] **Co@NPCNFs/S**	38%	1 M $NaClO_4$+EC/DEC	0.1C	800	0.07% at 100th 0.038% at 800th	871, 683, 275, 154 mAh g^{-1} at 168, 838, 1675, 5025 mA g^{-1}
VO_2 nanoflowers wrapped by RGO[8] **RGO/VO_2**	40%	1 M $NaClO_4$ in TEGDME	2C	1000	0.07%	558.1, 308.1, 234, 194 mAh g^{-1} at 335, 838, 1675, 3350 mA g^{-1}

High-surface-area mesoporous carbon with nanocopper[9] **HSMC-Cu-S**	50%	1 M $NaClO_4$ in EC/DMC	0.03C	110	0.127%	700, 560, 440, 320, 100 mAh g^{-1} at 50.3, 101, 1005, 2010, 5025 mA g^{-1}
Double-shell carbon microspheres assembled by hollow carbon nanobeads[10] **PCMs-S**	34%	1M $NaClO_4$ in PC/EC + 5wt.% FEC	100 mA g^{-1}	350	0.17%	402, 205, 74, 57 mAh g^{-1} at 100, 200, 500, 2000 mA g^{-1}
Thioether bond functionalized carbon[11] **SC**	21.5%	1 M $NaClO_4$+PC+ 5wt.% FEC	100 mA g^{-1}	200	0.17%	500, 430, 405, 390, 345, 300 mA h g^{-1} at 100, 200, 400, 800, 1600, 3200 mA g^{-1}
Mesoporous carbon hollow nanospheres[12] **S@iMCHS**	46%	1 M $NaClO_4$+ PC/EC + 5 wt.% FEC	100 mA g^{-1}	200	0.056%	391, 386, 352, 305, 174, 127 mAh g^{-1} at 100, 200, 500, 1000, 2000, 5000 mA g^{-1}
Hollow carbon nanospheres with Fe on the surface[13] **S@Fe-HC**	40%	1 M $NaClO_4$+ PC/EC + 5 wt.% FEC	100 mA g^{-1}	1000	0.06%	820, 498, 383, 313, 269 and 220 mAh g^{-1} at 100, 200, 500, 1000, 2000, 5000 mA g^{-1}
Nitrogen-doped porous carbon nanotubes with nickel sulfide nanocrystals[14] **NiS_2@NPCTs/S**	56%	1 M $NaClO_4$ in EC/PC+ 3 wt.% FEC	1000 mA $g^{−1}$	750	0.078%	760, 691, 557, 457, 346, 203 mAh g^{-1} at 100, 200, 500, 1000, 2000, 5000 mA $g^{−1}$

Reference

[1] B. Guo, W. Du, T. Yang, J. Deng, D. Liu, Y. Qi, J. Jiang, S.-J. Bao, M. Xu. Nickel hollow spheres concatenated by nitrogen-doped carbon fibers for enhancing electrochemical kinetics of sodium-sulfur batteries. *Adv. Sci.* 1902617, doi: 10.1002/advs.201902617 (2019).

[2] I. Kim, C. H. Kim, S. H. Choi, J. P. Ahn, J. H. Ahn, K. W. Kim, E. J. Cairns, H. J. Ahn. A singular flexible cathode for room temperature sodium/sulfur battery. *J. Power Sources*, **307**, 31-37, doi: 10.1016/j.jpowsour.2015.12.035 (2016).

[3] R. Carter, L. Oakes, A. Douglas, N. Muralidharan, A. P. Cohn, C. L. Pint. A sugar-derived room-temperature sodium sulfur battery with long term cycling stability. *Nano lett.* **17**, 1863-1869, doi: 10.1021/acs.nanolett.6b05172 (2017).

[4] Q.-Q. Lu, X.-Y. Wang, J. Cao, C. Chen, K. Chen, Z.-F. Zhao, Z.-Q. Niu, J. Chen. Freestanding carbon fiber cloth/sulfur composites for flexible room-temperature sodium-sulfur batteries. *Energy Storage Mater.* **8**, 77, doi: http://dx.doi.org/10.1016/j.ensm.2017.05.001 (2017).

[5] Y. M. Chen, W. F. Liang, S. Li, F. Zou, S. M. Bhaway, Z. Qiang, M. Gao, B. D. Vogt, Y. Zhu. A nitrogen doped carbonized metal-organic framework for high stability room temperature sodium-sulfur batteries. *J. Mater. Chem. A.* **4**, 12471-12478, doi: 10.1039/c6ta04529d (2016).

[6] T. Yang , B. Guo, W. Du, M. K. Aslam, M. Tao, W. Zhong, Y. Chen, S.-J.n Bao, X. Zhang, M. Xu. Design and construction of sodium polysulfides defense system for room-temperature Na-S battery. *Adv. Sci.* 1901557, doi: 10.1002/advs.201901557 (2019).

[7] W. Du, W. Gao, T. Yang, B. Guo, L. Zhang, S.-j. Bao, Y. Chen, M. Xu. Cobalt nanoparticles embedded into free-standing carbon nanofibers as catalyst for room-temperature sodium-sulfur batteries. *J. Colloid & Inter. Sci.* **565**, 63-69, doi: 10.1016/j.jcis.2020.01.010J (2020).

[8] W. Du, Y. Wu, T. Yang, B. Guo, D. Liu, S.-J. Bao, M. Xu. Rational construction of rGO/VO_2 nanoflowers as sulfur multifunctional hosts for room temperature Na-S batteries. *Chem. Eng. J.* **379**, 122359, doi: 10.1016/j.cej.2019.122359 (2020).

[9] S. Zheng, P. Han, Z. Han, P. Li, H. Zhang, J. Yang. Boosting performance of Na-S batteries using sulfur-doped $Ti_3C_2T_x$ MXene nanosheets with a strong affinity to sodium polysulfides. *Adv. Energy Mater.* 1400226, doi: 10.1021/acsnano.9b04977 (2014).

[10] L. Zhang, B. Zhang, Y. Dou, Y. Wang, M. Al-Mamun, X. Hu, H. Liu. Nano-copper-assisted immobilization of sulfur in high-surface-area mesoporous carbon cathodes for room temperature Na-S batteries. *ACS Appl. Mater. Inter.* **10**(24), 20422-20428, doi: 10.1002/aenm.201400226 (2014).

[11] K. J. Chen, H. J. Li, Y. Xu, K. Liu, H. M. Li, X. W. Xu, X. Q. Qiu, M. Liu. Self-assembling hollow carbon nanobeads into double-shell microspheres as a hierarchical sulfur host for sustainable room-temperature sodium-sulfur batteries. *Nanoscale.* **11**, 5967-5973, doi: 10.1021/acsami.8b03850 (2019).

[12] Y. -X. Wang, J. P. Yang, W. H. Lai, S. -L. Chou, Q. -F. Gu, H. K. Liu,,D. Y. Zhao, S. X. Dou. Achieving high-performance room-temperature sodium-sulfur batteries with S@interconnected mesoporous carbon hollow nanospheres. *J. Am. Chem. Soc.* **138**, 16576-16579, doi: 10.1021/jacs.6b08685 (2016).

[13] B.-W. Zhang, T. Sheng, Y.-X. Wang, S. Chou, K. Davey, S.-X. Dou, Shi-Zhang Qiao. Long-life room-temperature sodium-sulfur batteries by virtue of transition-metal-nanocluster-sulfur interactions. *Angew. Chem. Int. Ed.* **57**, 1-6, doi: 10.1002/anie.201811080 (2018).

[14] Z. Yan, J. Xiao, W. Lai, L. Wang, F. Gebert, Y. Wang, Q.Gu, H. Liu, S.-L. Chou, H. Liu, S.-X. Dou. Nickel sulfide nanocrystals on nitrogen-doped porous carbon

nanotubes with high-efficiency electrocatalysis for room-temperature sodium-sulfur batteries. *Nature Comm.* **10**, 4793, doi: 10.1038/s41467-019-11600-3 (2019).
